# Dr. Male's Case of Hepatitis Suppurans

**Published:** 1804-12-01

**Authors:** Geo. Edw. Male

**Affiliations:** Birmingham


### *To the Editors of the Medical and Physical Journal.*

Gentlemen,

THE disease of hepatitis suppurans terminating favourably, being a rare occurrence, but very few instances of which, if any, are upon record, I have transmitted to you the following case, and request you will give it a place in the Medical Journal.

W. B. aged twenty-three years, a private in the Loyal Birmingham Volunteers, when marching in a very hot day in June last, with the battalion, from Litchfield to Birmingham, was suddenly taken ill on the road, but continued his march. I saw him two days after his arrival here, when he complained of a pain in his right hypochondrium, extending to the scapula on the same side, with cough, and inability to lie on the left side, and every other symptom of acute hepatitis; his pulse was quick and full. I ordered him to be bled to ℥xvj. immediately, a large blister to be applied to the right hypochondrium, and a laxative medicine with calomel to be taken. The blood exhibited much of the inflammatory or buffy coat, but the symptoms were not much alleviated, and venesection was ordered to be repeated to the same quantity as before, and small doses of tartarized antimony to be taken frequently. The discharge from the blistered part was kept up by the application of the unguentum cantharidis.; subacid fruits, and low diet were enjoined. The day following, he found himself better, but not free from pain; the blood had the same appearance as before, and his pulse being still full and strong, he was again bled, after the interval of one day, to ℥xij. The blood still showed a slight inflammatory coat, and he found himself much relieved, the pain having left his shoulder, and being able to lie on his left side, but he still felt some uneasiness about the region of the liver; his appetite was bad, and his ancles became œdematous. He was now ordered to take small doses of calomel, morning and evening, and (the part where the blister was applied being healed) a drachm of the unguentum hydrargyri was directed to be rubbed into the side twice a day, and he took a mixture with about five drops of laudanum, three times a day. This was continued for a few days, when Dr. Carmichael attended the patient with me; his cough became more troublesome, and was followed by a purulent expectoration. On examining the affected side, a small tumour was observed with slight fluctuation, and the patient began to complain of cold shiverings, and soon afterwards became hectic. We had now no doubt but that suppuration of the liver was taking place, and a seton being passed through the tumour, more than a quart of very fetid matter immediately issued from it, and the expectoration of pus ceased: a mixture containing the nitric acid and camphorated tincture of opium was prescribed. The discharge from the seton continued the whole day. On the morning following, when we saw our patient, he was in a most deplorable situation; the thread of the seton not having been properly tied, had come out in the night, and the discharge from the side had stopped, but large quantities of fetid pus, of a coffee colour, mixed with blood, were emitted from the mouth; the cough was most urgent, the pulse weak and rapid; cold drops of sweat were distilled from every part of his body, and his cheeks were alternately overspread with a deadly paleness, or flushed with hectic fever, and a speedy dissolution was to be expected. Nearly two large chamber-pots full of pus were thus discharged from his mouth in about twelve hours. The thread was immediately replaced through the tumour, and poultices applied to the side, and small doses of opiates, with mucilages, &c. prescribed. In a few hours the discharge by the mouth ceased, and never returned; that from the side appeared again, and in considerable quantity. The day following the patient, though extremely weak, felt himself much easier, and the violence of the cough (though it troubled him for some time afterwards) was considerably abated. He continued the use of the above medicines with trifling alterations, for some time, which were afterwards changed for the decoction of bark with a few drops of laudanum. The hectic fever gradually left him, and the cough and pulmonary affection (produced by the discharge of the matter through the diaphragm and lungs to the mouth,) have now entirely disappeared; and after the space of three months, the whole of which time pus continued to issue from the affected part, he perfectly recovered his health, and is now as well as at any period of his life, excepting that his side is not yet quite healed.

On being present some time ago at the dissection of the body of a person who died of enteritis, evident marks of a former former suppuration of the stomach, (from which the patient had recovered,) were found. I may, perhaps, trouble you with the case at some future time. I am, &c.

*Birmingham, Sept.* 8, 1804. GEO. EDW. MALE, M. D.

